# Work-related psychosocial factors and working life expectancy among Finnish public sector employees aged 50 years or older

**DOI:** 10.5271/sjweh.4298

**Published:** 2026-05-01

**Authors:** Eija Haukka, Katriina Heikkilä, Jaana Pentti, Jussi Vahtera, Holendro Singh Chungkham, Paola Zaninotto, Mika Kivimäki, Jenni Ervasti, Sari Stenholm

**Affiliations:** 1 Finnish Institute of Occupational Health, Helsinki, Finland.; 2 Unit of Clinical Medicine, Faculty of Medicine, University of Oulu, Oulu, Finland.; 3 Department of Public Health, University of Turku and Turku University Hospital, Turku, Finland.; 4 Centre for Population Health Research, University of Turku and Turku University Hospital, Turku, Finland.; 5 Clinicum, Faculty of Medicine, University of Helsinki, Helsinki, Finland.; 6 Department of Epidemiology and Public Health, University College London, London, United Kingdom.; 7 Psychobiology and Epidemiology Division, Department of Psychology, Stockholm University, Stockholm, Sweden.; 8 Brain Sciences, University College London, London, United Kingdom.; 9 Research Services, Turku University Hospital and University of Turku, Turku, Finland.

**Keywords:** effort–reward imbalance, cohort study, job control, job demand, job strain, labor force participation, older worker, organizational justice, procedural justice, register data, relational justice, work participation, working years

## Abstract

**Objectives:**

This study aimed to examine the associations between work-related psychosocial factors and working life expectancy (WLE) across occupational groups among Finnish public sector employees aged ≥50 years.

**Methods:**

In this cohort study, 70 662 Finnish public sector employees completed surveys on work-related psychosocial factors in 2000–2002, 2004, 2008, 2011–2012, 2013–2014, and 2015–2016, with each participant responding at least once at age ≥50 years (response rates 66–71%; 80% female). Survey data were linked to pensionable earnings records to verify work participation until 31 December 2018. WLE between ages 50 and 68 years was estimated using a multi-state life tables approach. Analyses were conducted among three occupational groups: managers and specialized professionals, non-manual professionals, and service and manual workers.

**Results:**

The overall WLE at age 50 was 13.1 years [95% confidence interval (CI) 13.1–13.2]. Work-related psychosocial factors were associated with shorter WLE across all occupational groups, with WLE shortening from the highest to the lowest occupational group. High effort–reward imbalance (ERI) was associated with the shortest WLE, approximately five months shorter than among employees with low ERI. Compared with managers and specialized professionals with low psychosocial risks, high ERI, high job strain, high relational or procedural injustice were each associated with an approximately 1-year shorter WLE among service and manual workers. Occupational group showed a stronger association with WLE than the accumulation of psychosocial risk factors. No sex differences in WLE were observed.

**Conclusions:**

These findings suggest that promoting favorable psychosocial working conditions may extend working careers and reduce inequalities in working life participation, particularly among service and manual workers.

Declining birth rates, increasing life expectancies, and subsequent growth of the ageing population have significantly raised the old-age dependency ratio, which was 34% in 2024 in the European Union (EU), and it is projected to reach 60% by 2100 (1). To tackle the challenge of decreasing numbers of workers, many countries have proposed policy measures such as raising the statutory retirement age and restricting pension eligibility. Despite longer life expectancy, the average duration of labor force participation remains relatively short (2). In 2024, the average working life expectancy (WLE) at age 15 in Europe was 37 years (3).

Poor health and reduced work ability are the most important reasons for early exit from paid employment (4). Nearly half of the EU population aged 55–64 reports a longstanding illness or health problem highlighting the scale of this challenge (5). As the workforce ages, a key concern is the accelerated decline in work ability after the age of 50 (6), which increases vulnerability to health-related labor market exit.

Work-related psychosocial factors are important determinants of health and work ability. Exposure to adverse psychosocial working conditions has been associated with an increased incidence of musculoskeletal (7) and mental disorders (8, 9), cardiovascular diseases (8, 9), and type 2 diabetes (10, 11). These health conditions, in turn, increase the likelihood of exiting employment prematurely.

Among psychosocial stressors, a high effort–reward imbalance (ERI) has been consistently associated with adverse labor market outcomes. High ERI, both at the work unit and individual levels has been associated with an increased risk of disability pensions due to depression, and individual-level ERI also with an increased the risk of musculoskeletal diseases (12). Further evidence (13) shows that ERI is negatively associated with self-rated work ability and increases intentions to apply for a disability pension. A six-year Swedish longitudinal study found that poor health, reduced work ability, and high ERI levels were associated with preferences for early retirement (14). Other psychosocial working conditions show similar patterns. Job strain has been identified as a risk factor for all-cause disability pension and disability pension due to musculoskeletal disorders, depression and coronary heart disease (15). Of the components of job strain, low job control has been linked to disability pension (16), early retirement (17) and unemployment (18), and high job demands to disability pension (19) and early retirement (20). Organizational injustice has been linked to higher risk of disability pension due to depression and musculoskeletal diseases (21). ERI, job strain, and organizational injustice often co-occur and increase the likelihood of disability pension compared to individuals not exposed to these stressors (22).

Despite the evidence linking work-related psychosocial factors to work ability and different exit routes from employment, limited research exists on their associations with WLE, which is a comprehensive summary measure of total work participation indicating the number of years a person at a given age can expect to work (23). Unlike studies focused on single exit routes and estimating risks for example for unemployment, retirement, or death, WLE accounts all the time in paid work and considers periods when individuals are not working due to unemployment or retirement and later return to work, either part- or full-time.

We identified only two studies that have examined the associations between work-related psychosocial factors and WLE. A study in Swedish employees (24) reported that job strain shortened WLE by six months to one year among individuals aged ≥50 years, compared to those not experiencing job strain. However, in that study, main analysis focused on the associations between job strain and WLE by sex and work status (full-/part-time), and to a lesser extent to occupational group, which is also important determinant of WLE. Another study (25) used a job exposure matrix (JEM) to assess job control in association with WLE among Swedish men and women. At age 30, men in low-control jobs had a 2.5-year shorter WLE compared to those in high-control jobs, while the difference for women was nearly five years. At age 40, the gap was 2.1 years for both sexes, decreasing to 1.1 years at age 50 and to approximately three months by age 60. The limitation with the JEM is that it does not capture individual variation within the same occupation, making it difficult to distinguish whether the observed association is due to job control or occupational group (26). We are not aware of studies that examined the associations between ERI, relational or procedural justice and WLE.

In this cohort study, we examined the associations of ERI, job strain, and relational and procedural justice and their accumulation with WLE among Finnish public sector employees aged ≥50 years, stratified by occupational group and sex.

## Methods

### Study population

The Finnish Public Sector (FPS) study is a dynamic cohort study of public sector personnel in Finland, established in 1997 (27). Information from surveys conducted in the years 2000–2002, 2004, 2008, 2011–2012, 2013–2014, and 2015–2016 was used (response rate 66–71% across the surveys), during which 156 321 participants responded to the survey at least once while working. The follow-up for each participant began when they responded to a survey the first time when aged 50–<68 years. For the current analysis, we included individuals who had data on any of the work-related psychosocial factors and covariates and were ≥50 years (N=70 662). The follow-up was based on data from the Earnings and Accrual Register maintained by the Finnish Centre for Pensions available until 31 December 2018. Follow-up continued until the age of 68, which at the time was the upper age limit for accruing earnings-related pension.

Helsinki and Uusimaa Hospital District ethics committee approved the FPS (HUS/1210/2016).

### Work-related psychosocial factors

Information on work-related psychosocial factors was obtained from the first survey the participant responded to after turning 50. Similarly, information on occupation was obtained at the time of the first survey. Psychosocial factors were divided into groups based on the overall distribution of the variables across all survey years combined.

*Effort–reward imbalance (ERI)* was measured using four survey questions (one on efforts and three on rewards) adapted from Siegrist's standard 10-item ERI scale (28, 29). Responses were given on a 5-point scale from 5=strongly agree to 1=strongly disagree. The ratio of the effort score and the mean of the self-reported reward scores formed the individual-level ERI, which was further divided into quartiles. The lowest quartile indicates lowest ERI and highest quartile highest ERI.

*Job control* was measured using a modified version of Karasek’s Job Content Questionnaire (JCQ) (30, 31). The job control scale combines two concepts: skill discretion (the opportunities of an individual to develop his or her special abilities within the job, six items) and decision authority (individual’s abilities to be part of the decision-making process within the organization, three items). These subscales were combined for the analysis. Responses were given on a 5-point scale from 5=strongly agree to 1=strongly disagree. After calculating the mean score of all items, job control was divided into quartiles so that lowest quartile indicates lowest job control and highest quartile highest job control.

*Job demands* were also measured with modified JCQ. The job demand scale consists of five items, which considered time pressures and deadlines, lack of time to do what was expected, and work overload. Responses were given on a 5-point scale from 5=strongly agree to 1=strongly disagree. After calculating the mean score of all items, job demands were divided into quartiles so that lowest quartile indicates lowest job demands and highest quartile highest job demands.

*Job strain* was defined as high demands (higher than median score, ie, two upper quartiles) and low control (lower than median score, ie, two lower quartiles); all other combinations of job demands and job control indicated no strain.

*The relational justice* scale (six items) (32, 33), assesses the quality of treatment employees experience in their interpersonal interactions during the completion of organizational processes. The scale includes items, such as whether the supervisors use kindness and consideration, are truthful, and can suppress personal biases. The response format was a 5-point scale from 5=strongly agree to 1=strongly disagree. After calculating the mean score of all items, relational justice was divided into quartiles so that the lowest quartile indicates the lowest relational justice and the highest quartile the highest relational justice.

*The procedural justice* scale (seven items) (32, 33), considers whether the decision-making procedures at the workplace are accurate, correctable, consistently applied, and whether the procedures include opinions from the people involved. Response format was a 5-point scale from 5=strongly agree to 1=strongly disagree. After calculating the mean score of all items, procedural justice was divided into quartiles so that the lowest quartile indicates the lowest procedural justice and the highest quartile the highest procedural justice.

The accumulation of work-related psychosocial risk factors (highest quartile for ERI and job strain, lowest quartile for relational and procedural justice) was defined as the number of these risk factors (0, 1, 2 or 3–4).

### Working life expectancy

Labor force participation during the follow-up was ascertained utilizing linked data from the Finnish Centre for Pensions' Earnings and Accrual Register, which contains data on all Finnish residents’ earnings (eg, wages and salaries from employment, self-employment, and social security benefits that accrue pension benefits) (34). The register does not contain specific information on sickness absence. Working and not working states were ascertained from dates of beginning and ending spells of employment in the register, while death was established from the Statistics Finland population data on mortality up to 31 December 2018. All participants were working at baseline and have complete data on all covariates.

### Covariates

Age, sex (men versus women) and occupation were obtained from the employers’ records. Data on occupational titles at the time of the first survey were converted to International Standard Classification of Occupations (ISCO) (35). Occupational groups were categorized into three groups: (i) managers and specialized professionals (ISCO classes 1–2), (ii) non-manual professionals (ISCO classes 3–4), and (iii) service and manual workers (ISCO classes 5–9).

### Statistical analysis

WLE between ages 50 and 68 years was estimated utilizing a multi-state life tables approach, based on transition probabilities between three states (working, not working and dead, see the supplementary material, www.sjweh.fi/article/4298, figure S1). To avoid fragmentation in outcome estimates due to frequent work status changes eg, short breaks between job contracts, we aggregated the data into three-month spells beginning on each individuals’ baseline date. We defined individuals as being at work if they spent any amount of time working during a spell and not working if they spent no time working. Participants were followed from the response date of first survey measurement until the first of the following occurred: age 68 years, death, or the end of the register follow-up on 31 December 2018. Each participant could be in one state at a time and transition between the working and not working states, death being defined as an absorbing state.

We used package *msm* [for multi-state survival models in panel data (36)] to predict transition probabilities across the three states using continuous time and *elect* (utilizing a Gompertz model to estimate mortality rates, which also incorporates age as time-dependent in the model) (37) to estimate WLE (23) in *R* (https://www.r-project.org). The WLE predictions at age 50 were obtained for each category of the work-related psychosocial factors and for their accumulation by sex and occupational group. In addition, WLE results only by occupational group were provided. Finally, we calculated 95% confidence intervals (CI) for the differences in WLE using parametric Monte-Carlo simulation-based uncertainty estimation procedure within the *elect* package in *R* (37). This procedure relies on 500 repeated simulations based on asymptotic properties of Maximum likelihood estimator for the multi-state models.

## Results

The baseline characteristics of participants by sex and occupational group are presented in table 1. The mean age of the participants at baseline was 54 years, and 80% were women. Service and manual workers had higher levels of high ERI (26%) and job strain (34%) compared to 18% and 11% among managers and specialized professionals. Relational and procedural justice varied less between the occupational groups. Furthermore, 24% of the respondents reported at least two work-related psychosocial risk factors (27% among service and manual workers versus 18% among managers and specialized professionals). Compared to the eligible population, women (80% versus 78%) and managers and specialized professionals (34% versus 33%) were slightly over-represented in the current study population.

**Table 1:** Baseline characteristics of the study participants by sex and occupational groups. Finnish public sector employees. [ERI=effort–reward imbalance; SD=standard deviation; Q=quartile.]

		Total (N=70 662) ^a^		Men						Women				
				Managers and specialized professionals (N=6179) ^b^		Non-manual professionals (N=3080) ^c^		Service and manual workers (N=4943) ^d^		Managers and specialized professionals (N=17 851) ^e^		Non-manual professionals (N=17 289) ^f^		Service and manual workers (N=21 320) ^g^
		N (%)		N (%)		N (%)		N (%)		N (%)		N (%)		N (%)
ERI														
	Q1 (low)	9118 (20.5)		1092 (28.5)		415 (22.5)		712 (22.1)		2212 (21.1)		2047 (18.3)		2640 (19.0)
	Q2	12 999 (29.2)		1264 (33.0)		473 (25.7)		818 (25.3)		3338 (31.8)		3306 (29.5)		3800 (27.4)
	Q3	12 295 (27.6)		908 (23.7)		509 (27.7)		874 (27.1)		2987 (28.4)		3160 (28.2)		3857 (27.8)
	Q4 (high)	10 074 (22.7)		565 (14.8)		444 (24.1)		824 (25.5)		1971 (18.8)		2700 (24.1)		3570 (25.7)
Job strain														
	No	53 881 (76.3)		5591 (90.5)		2542 (82.5)		3776 (76.4)		15 784 (88.4)		12 728 (73.6)		13 460 (63.1)
	Yes	16781 (23.8)		588 (9.5)		538 (17.5)		1167 (23.6)		2067 (11.6)		4561 (26.4)		7860 (36.9)
Relational justice														
	Q1 (high)	15 751 (22.8)		1448 (23.7)		564 (18.6)		830 (17.4)		4627 (26.1)		3474 (20.4)		4808 (23.6)
	Q2	17 568 (25.4)		1689 (27.6)		766 (25.2)		1194 (25.0)		4544 (25.6)		4103 (24.0)		5272 (25.9)
	Q3	18 967 (27.4)		1654 (27.1)		858 (28.2)		1383 (29.0)		4722 (26.6)		4871 (28.5)		5479 (26.9)
	Q4 (low)	16 830 (24.4)		1319 (21.6)		851 (28.0)		1367 (28.6)		3832 (21.6)		4624 (27.1)		4837 (23.7)
Procedural justice														
	Q1 (high)	16 508 (23.9)		1925 (31.5)		630 (20.7)		902 (18.9)		4852 (27.4)		2832 (16.6)		5367 (26.3)
	Q2	17 879 (25.9)		1652 (27.0)		831 (27.3)		1175 (24.6)		4447 (25.1)		4281 (25.1)		5493 (26.9)
	Q3	17 427 (25.2)		1179 (19.3)		716 (23.6)		1289 (27.0)		4135 (23.3)		4872 (28.5)		5236 (25.7)
	Q4 (low)	17 302 (25.0)		1354 (22.2)		862 (28.4)		1408 (29.5)		4291 (24.2)		5087 (29.8)		4300 (21.1)
Work-related psycho-social risk factors														
	0	33 250 (47.4)		3676 (59.9)		1451 (47.5)		2142 (43.9)		9909 (55.7)		7221 (42.0)		8851 (42.1)
	1	20 043 (28.6)		1433 (23.3)		841 (27.5)		1377 (28.2)		4661 (26.2)		5195 (30.2)		6536 (31.1)
	2	10 884 (15.5)		750 (12.2)		497 (16.3)		809 (16.6)		2322 (13.1)		3003 (17.5)		3503 (16.7)
	3–4	5937 (8.5)		283 (4.6)		268 (8.8)		549 (11.3)		897 (5.0)		1795 (10.4)		2145 (10.2)

^a^ Mean age 54.1 (SD 3.5) years. ^b^ Mean age 54.5 (SD 3.8) years. ^c^ Mean age 54.3 (SD 3.7) years. ^d^ Mean age 54.3 (SD 3.4) years. ^e^ Mean age 54.0 (SD 3.5) years. ^f^ Mean age 53.8 (SD 3.4) years. ^g^ Mean age 54.3 (SD 3.5) years.

**Figure 1:**
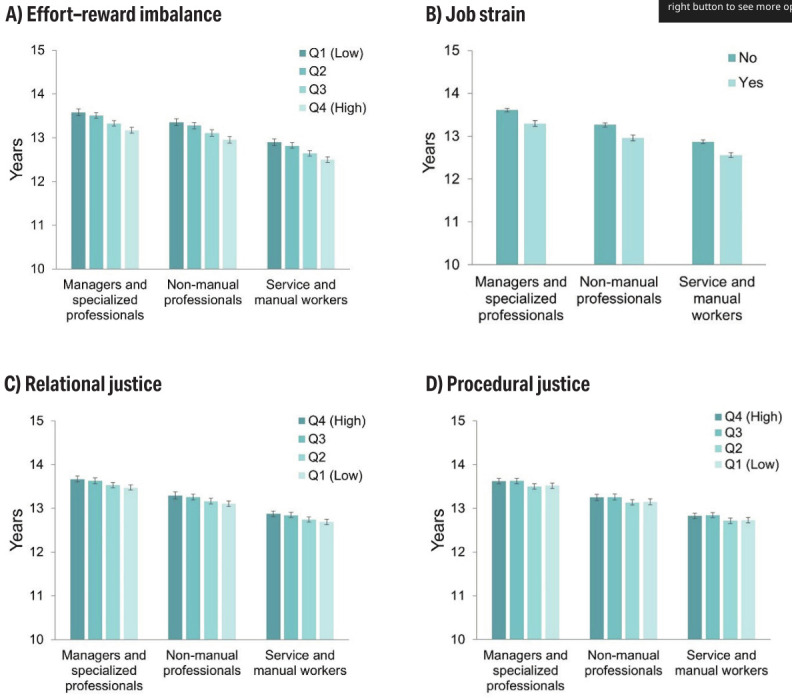
Estimated working life expectancies at age 50 among Finnish public sector employees aged 50–68 (N=70 662), by work-related psychosocial factors and occupational groups. [Q=quartile.]

The overall WLE at age 50 was 13.1 years (95% CI 13.1–13.2), and WLE varied from 12.8 years among service and manual workers to 13.6 years among managers and specialized professionals. Figure 1 illustrates the estimated WLE at age 50 based on ERI, job strain, relational and procedural justice across occupational groups. Psychosocial risk factors were associated with shorter WLE across all occupational groups, with WLE shortening from managers and specialized professionals to service and manual workers.

Except for ERI, the associations were rather similar regardless of occupational group. Among male managers and specialized professionals, those with low ERI had a 4.6-month longer WLE compared to those with high ERI. The differences were 4.2 months among non-manual professionals and 4.3 months among service and manual workers. For women, the corresponding figures were 5.1, 4.8, and 4.9 months. For job strain the difference between those without and with job strain were 3.7, 3.6, and 3.6 months among men and 3.8, 3.7, and 3.8 months among women, respectively. In relational injustice, smaller differences were observed between low and high injustice groups across all occupational groups: 2.2, 2.0, and 2.0 months for men, and 2.4, 2.2, and 2.2 months for women. For procedural injustice, the difference between high and low injustice groups was only 1.2 months across all occupational groups for both sexes, which was not statistically significant. (supplementary table S2.)

WLE results for two dimensions of job strain, ie, job control and job demands, are presented supplementary figure S2 and table S3. The results for the separate dimensions were consistent with those for the combined job strain variable.

Compared to managers and specialized professionals and the lowest levels of psychosocial risk factors, service and manual workers who reported job strain or experienced high levels of ERI, relational, or procedural injustice had a one-year shorter WLE. The longest WLE (13.7 years) was observed among managers and specialized professionals with high relational justice, while the shortest (12.5 years) was found among service and manual workers with high ERI. No differences in WLE between the sexes were observed (table 2).

**Table 2:** Estimated working life expectancies (WLE in years) at age 50 among Finnish public sector employees of age 50–68 (N=70 662), by individual and number of work-related psychosocial risk factors, sex and occupational group. [CI=confidence interval; Q=quartile.]

		Men									Women							
		Managers and specialized professionals			Non-manual professionals			Service and manual workers			Managers and specialized professionals			Non-manual professionals			Service and manual workers	
		WLE	95% CI		WLE	95% CI		WLE	95% CI		WLE	95% CI		WLE	95% CI		WLE	95% CI
Effort-reward imbalance																		
	Q1 (low)	13.5	13.4–13.6		13.3	13.1–13.4		12.8	12.7–12.9		13.6	13.5–13.7		13.4	13.3–13.3		12.9	12.9–13.0
	Q2	13.5	13.4–13.5		13.2	13.1–13.3		12.8	12.8–12.9		13.5	13.4–13.6		13.3	13.2–13.4		12.8	12.8–12.9
	Q3	13.3	13.2–13.4		13.0	12.9–13.1		12.6	12.5–12.7		13.3	13.3–13.4		13.1	13.0–13.2		12.7	12.6–12.7
	Q4 (high)	13.1	13.0–13.2		12.9	12.8–13.0		12.5	12.3–12.5		13.2	13.1–13.3		13.0	12.9–13.0		12.5	12.4–12.6
Job strain																		
	No	13.6	13.5–13.7		13.2	13.2–13.3		12.9	12.8–12.9		13.6	13.6–13.7		13.3	13.2–13.3		12.9	12.8–12.9
	Yes	13.3	13.2–13.4		13.0	12.9–13.0		12.6	12.5–12.6		13.3	13.2–13.4		13.0	13.0–13.0		12.6	12.5–12.6
Relational justice																		
	Q4 (high)	13.7	13.6–13.8		13.4	13.2–13.4		12.9	12.8–13.0		13.7	13.6–13.7		13.3	13.2–13.4		12.9	12.8–12.9
	Q3	13.7	13.6–13.7		13.3	13.2–13.4		12.9	12.8–13.0		13.6	13.6–13.7		13.3	13.2–13.3		12.8	12.8–12.9
	Q2	13.6	13.5–13.6		13.2	13.1–13.3		12.8	12.7–12.9		13.5	13.5–13.6		13.2	13.1–13.2		12.7	12.7–12.8
	Q1 (low)	13.5	13.4–13.6		13.1	13.0–13.2		12.7	12.6–12.8		13.5	13.4–13.5		13.1	13.0–13.2		12.7	12.6–12.8
Procedural justice																		
	Q4 (high)	13.6	13.6–13.7		13.2	13.1–13.4		12.8	12.7–12.9		13.6	13.6–13.7		13.3	13.2–13.3		12.8	12.8–12.9
	Q3	13.6	13.6–13.7		13.3	13.2–13.4		12.9	12.8–13.0		13.6	13.6–13.7		13.3	13.2–13.3		12.8	12.8–12.9
	Q2	13.5	13.4–13.6		13.2	13.1–13.3		12.8	12.7–12.8		13.5	13.4–13.5		13.1	13.1–13.2		12.7	12.7–12.8
	Q1 (low)	13.5	13.4–13.6		13.1	13.0–13.2		12.7	12.7–12.8		13.5	13.5–13.6		13.2	13.1–13.2		12.7	12.7–12.8
Number of work-related psychosocial risk factors																		
	0	13.7	13.6–13.7		13.3	13.2–13.4		12.9	12.8–13.0		13.7	13.6–13.7		13.3	13.3–13.4		12.9	12.8–13.0
	1	13.5	13.5–13.6		13.2	13.2–13.3		12.8	12.7–12.9		13.5	13.4–13.6		13.2	13.1–13.2		12.8	12.7–12.8
	2	13.4	13.3–13.5		13.0	13.0–13.2		12.7	12.6–12.8		13.4	13.3–13.5		13.1	13.0–13.2		12.7	12.6–12.7
	3–4	13.2	13.1–13.3		12.9	12.8–13.0		12.5	12.4–12.6		13.2	13.1–13.3		12.9	12.8–13.0		12.5	12.4–12.6

**Figure 2:**
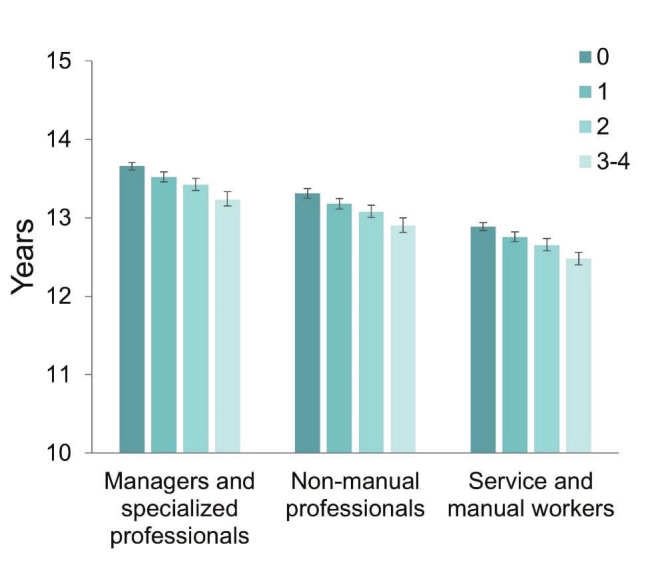
Estimated working life expectancies at age 50 among Finnish public sector employees aged 50–68 (N=70 662), by number of work-related psychosocial risk factors and occupational groups.

When examining the accumulation of work-related psychosocial risk factors, an increasing number of risk factors was associated with a lower WLE within each occupational group (figure 2). Notably, service and manual workers with no psychosocial risk factors had shorter WLE than managers and specialized professionals with 3–4 risk factors (12.9 versus 13.2 years).

## Discussion

We analyzed the association between work-related psychosocial factors and WLE between ages 50 and 68 years among Finnish public sector workers. Within each occupational group, employees with high ERI had a WLE about 4–5 months shorter than those reporting low ERI. In addition to high ERI, high job strain, and high relational or procedural injustice were each linked to shorter WLE across all occupational groups, with WLE shortening from the highest to the lowest occupational group. Employees in service and manual occupations and the highest levels of each psychosocial risk factors had one-year shorter WLE compared to managers and specialized professionals and the lowest risk levels. The shortest WLE (12.5 years) was observed among employees in service and manual occupations and high ERI, and the longest (13.7 years) among managers and specialized professionals and high relational justice. Importantly, service and manual workers without psychosocial risk factors still had shorter WLE than managers and specialized professionals with 3–4 psychosocial risk factors, indicating the decisive influence of occupational group on the WLE. No differences in the WLE were observed between sexes.

To our knowledge, this is the most comprehensive study to date examining the associations between multiple work-related psychosocial factors and WLE over up to 18 years of follow-up in a sample of more than 70 000 employees. Overall, only a few previous studies have investigated the relationships between psychosocial work factors and WLE, and comparison of results is complicated due to differences in exposure or outcome definitions as well as methods used.

Our findings on job strain, align with those of a study among Swedish employees (24), that examined the association between job strain and WLE among 13 225 individuals aged 50–75 years. Similarly to our study, they applied a multi-state model and assessed the exposure based on Karasek’s job demand–control model. In their primary sex-stratified analysis, job strain was associated with a shorter WLE across all measures – total WLE, as well as WLE in full- and part-time employment – for both men and women. Individuals experiencing job strain had approximately six months to one-year shorter WLE compared to those without job strain. As supplementary results, they presented WLE by occupational group. Occupation was based on self-reported job title, coded according to the Swedish socioeconomic classification, and categorized as professional, intermediate, and routine groups. In all occupational groups, WLE was six months shorter among those who experienced job strain compared to those who did not for both men and women. In our study, the corresponding difference was approximately four months for both sexes. Furthermore, the study observed that, among individuals in routine occupations with job strain, WLE was about 1.5 years shorter compared to those in professional occupations without job strain, regardless of sex. In our study, the corresponding difference was one year. These short differences in WLE may partly be explained by the length of the follow-up periods. The Swedish study followed individuals up to age 75, whereas our analysis was limited to age 68, which was the retirement age in Finland at that time. A longer follow-up period increases the likelihood of capturing the cumulative effects of job strain, as continuation in employment at older ages is more sensitive to health and working conditions. This may lead to larger differences in WLE in studies with extended follow-up.

Another Swedish study (25), based on register data on a random sample of 100 000 individuals, investigated how job control relates to WLE at ages 30, 40, 50, and 60 over a 15-year period. At the age of 30, high job control was associated with longer WLE: nearly five additional years for women and two and half years for men. However, this association weakened with age. By the age of 50, the difference between high and low job control jobs was only about one year for both sexes, and at the age of 60, the difference was reduced to just three months. In our study, managers and specialized professionals with high job control WLE at age 50 was one year longer compared to those in service and manual occupations with low job control, and seven months longer compared to those service and manual occupations with high job control. The results are consistent, even though job control was measured differently. Swedish researchers used JEM, which offers an objective measure and an average estimate of exposure for a given occupation but does not account for individual variation within the same occupation. In contrast, we relied on individual-level self-reported job control, which may be subject to recall bias but captures the employee’s own experience rather than relying on occupational averages. Perceived job control also reflects organizational culture and supervisory practices that objective matrices cannot capture, and it provides up-to-date information on actual job control. We primarily used job strain as exposure but also analyzed the dimensions of job control and demands separately, which were consistent with the results for job strain.

Schram et al (38) examined 11 800 Dutch workers aged 50–66 years and found that, at the age of 50, men and women with a low educational level had a four- and five-month shorter WLE, respectively, compared to those with a high educational level. Their study explored associations with poor working conditions but focused more on the number of working years lost (WYL) due to premature exit from the labor market than on total WLE. They concluded that workers exposed to unfavorable working conditions – combining both physical workload and high psychological and emotional demands, low autonomy, and poor social support – had up to 0.63 years more WYL due to involuntary pathways than workers with favorable working conditions. Psychosocial risk factors were associated with greater WYL than physical workload, particularly low social support.

We found no previous studies that focused specifically on the associations of ERI, relational justice, or procedural justice with WLE. We observed that ERI was most strongly associated with WLE across all occupational groups and for both sexes when comparing those with low versus high ERI, followed by job strain and relational justice. Low ERI has been shown to increase in importance with age for continued work among employees aged ≥59 years (39). Given that ERI, job strain, and organizational justice tend to cluster in the same individuals and increase the risk of work disability (22), their combined presence may likely have a substantial negative association on WLE. This suggests that cumulative exposure to these psychosocial stressors may shorten working careers by increasing the likelihood of early exit from the labor market. However, we observed that occupational group had a stronger association with WLE than the accumulation of psychosocial risk factors.

In Finland, the overall WLE for men and women is very similar (3), and our findings among public sector employees align with this pattern. We observed no sex differences in the associations between psychosocial risks and WLE. Although men are less represented in the public sector, they often work in similar roles (eg, as teachers, in personnel and financial administration, and in expert positions) as women, which means their exposure to ERI, job strain, and organizational injustice is likely comparable. Job organization, leadership practices, and reward structures are largely uniform, which may further reduce sex differences in these associations.

### Strengths and limitations

The study’s strengths include a large sample size, a longitudinal design with up to 18 years of register-based follow-up on work participation, analysis of the associations of several work-related psychosocial factors with WLE both by occupational group and sex, and utilization of a multi-state model approach in estimation of WLE. The multi-state model is a robust method for capturing processes and transitions where individuals move between multiple states over time. Unlike traditional survival models that focus on a single endpoint (eg, unemployment, death), this approach accommodates intermediate events and recurrent transitions, providing a comprehensive measure of total work participation.

The limitations of the study stem from the exposure ascertainment. Self-report is the only way to measure work-related psychosocial factors, but self-reported measures may be subject to recall and/or social desirability biases and fluctuations in momentary mood. The inclusion period for the study was long, spanning 16 years, during which working life may have evolved. However, our additional analyses suggest that the prevalence of psychosocial risk factors remained relatively constant across the baseline years (supplementary table S3). Survey-based studies typically involve selective participation, and in our study women and managers and specialized professionals were only slightly over-represented. Moreover, the sample consists exclusively of public sector employees, which restricts the generalizability of the findings to other sectors or to self-employed individuals, where job conditions, pay structures, employment security, and psychosocial work factors may differ substantially.

### Concluding remarks

This study showed that, among work-related psychosocial factors, high ERI was associated with shortest WLE across all occupational groups by approximately 4–5 months. Employees in service and manual occupations who were exposed to high psychosocial work stressors – such as ERI, job strain, and relational or procedural injustice – had, on average, one-year shorter working life compared to managers and specialized professionals with lower work-related psychosocial exposure. The accumulation of psychosocial risk factors was also associated with shorter WLE, although less strongly than low occupational group. The results did not differ between men and women. These findings suggest that promoting favorable psychosocial working conditions among employees in service and manual occupations may represent one potential mean for extending working careers and reduce inequalities in working life participation with advancing age.

## Supplementary material

Supplementary material

## Data Availability

The anonymized questionnaire data used in this study can be made available upon request to the study team. Linked register data on labor force participation and mortality require separate permission from the Findata, the Health and Social Data Permit Authority in Finland.
