# Supplementary material for: Work-related psychosocial factors and working life expectancy among Finnish public sector employees aged 50 years or older
Source: Scand J Work Environ Health. 2026 Apr 30;52(3):272–81. doi: 10.5271/sjweh.4298 (PMC13182574; doi:10.5271/sjweh.4298)
Supplement: Supplementary material [file SJWEH-52-272-S001.pdf]

Work-related psychosocial factors and working life expectancy among Finnish public sector employees age 50 years or more[1]

by Eija Haukka, PhD, Katriina Heikkilä, PhD, Jaana Pentti, BSc, Jussi Vahtera, MD, PhD, Holendro Singh Chungkham, PhD, Paola Zaninotto, PhD, Mika Kivimäki, PhD, Jenni Ervasti, PhD, Sari Stenholm, PhD[2]

1. SUPPLEMENTARY MATERIAL
2. Correspondence to: Dr. Sari Stenholm, University of Turku, Department of Public Health FI-20014 University of Turku, Finland. [E-mail: sari.stenholm@utu.fi]

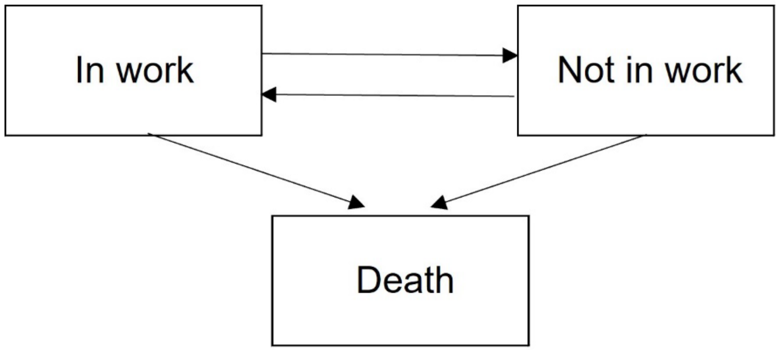

Supplementary Figure S1. The three-state model of working life expectancy (WLE) estimation.

a) Job control **b) Job demands**

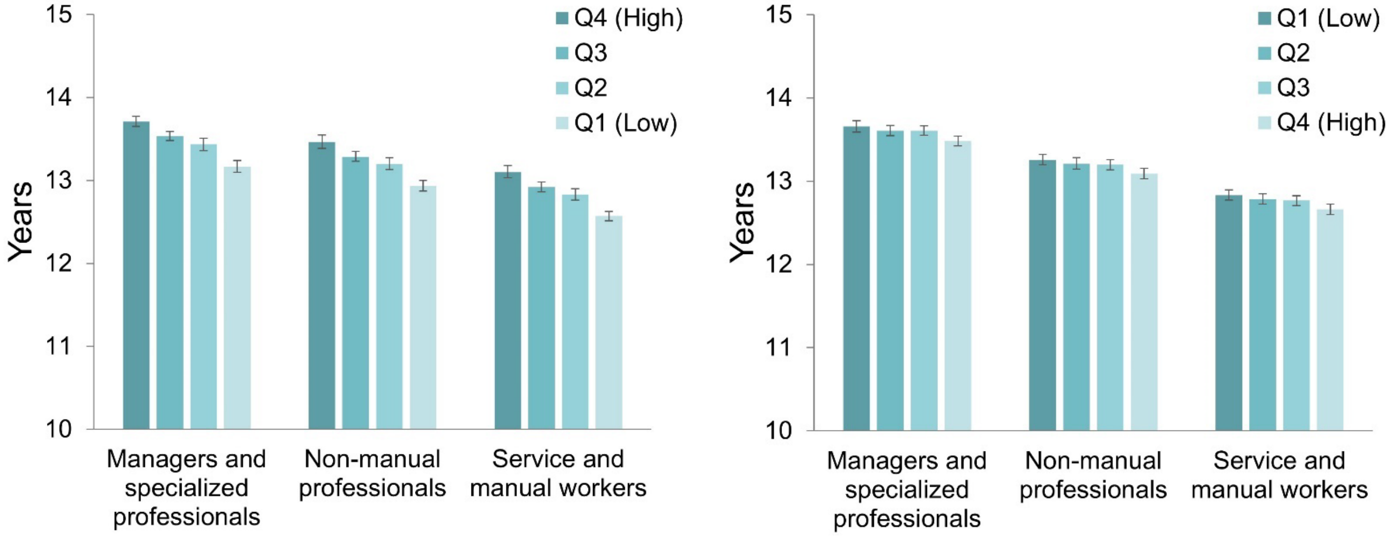

Supplementary Figure S2. Estimated working life expectancies at age 50 among Finnish public sector employees of age 50 to 68 (n=70, 662), for a) job control and b) job demands by occupational groups. [Q= quartile.]

Supplementary Table S1. Distribution of work-related psychosocial risk factors across all baseline years.

	Baseline year					
	2000-02 N=16170 %	2004 N=8557 %	2008 N=10513 %	2011-12 N=10506 %	2013-14 N=16369 %	2015-16 N=8547 %
Total	22.9	12.1	14.9	14.9	23.2	12.1
Effort-reward imbalance						
Q1 (Low)	19.1	19.4	22.7	21.2	N/A	N/A
Q2	28.5	29.1	29.5	29.8	N/A	N/A
Q3	28.2	29.0	27.1	26.3	N/A	N/A
Q4 (High)	24.2	22.5	20.8	22.7	N/A	N/A
Job strain						
No	75.4	75.4	77.0	78.0	75.3	76.9
Yes	24.6	24.6	23.0	22.0	24.7	23.2
Relational justice						
Q4 (High)	18.4	21.2	22.4	24.3	25.3	27.4
Q3	25.1	24.9	25.9	25.4	25.7	25.3
Q2	29.9	28.9	27.2	26.6	26.0	25.2
Q1 (Low)	26.6	25.0	24.6	23.9	23.0	22.2
Procedural justice						
Q4 (High)	22.4	22.5	25.7	24.4	24.0	25.1
Q3	26.6	27.0	25.6	25.6	25.1	25.8
Q2	25.2	25.5	26.1	25.4	24.7	24.2
Q1 (Low)	25.8	25.1	22.6	24.6	26.1	24.9

Supplementary Table S2. Comparison of working life expectancies (WLE, months) at age 50 among Finnish public sector employees of age 50 to 68 (n=70, 662), by work-related psychosocial factors, sex and occupational group. [CI= confidence interval.]

	Occupational group					
	Managers and specialized professionals		Non-manual professionals		Service and manual workers	
**Comparison**	**WLE**	**95% CI**	**WLE**	**95% CI**	**WLE**	**95% CI**
Men						
Effort-reward imbalance: Low vs. high	4.6	2.9-6.2	4.2	2.3-6.1	4.3	2.5-6.0
Job strain: No vs. yes	3.7	2.2-5.1	3.6	2.1-5.1	3.6	2.3-4.9
Relational justice: High vs. low	2.2	0.9-3.6	2.0	0.3-3.7	2.0	0.5-3.6
Procedural justice: High vs. low	1.2	-0.1-2.6	1.2	-0.5-3.0	1.2	-0.3-2.8
Women						
Effort-reward imbalance: Low vs. high	5.1	3.8-6.3	4.8	3.6-6.1	4.9	3.8-6.1
Job strain: No vs. yes	3.8	2.8-4.8	3.7	2.8-4.7	3.8	2.9-4.6
Relational justice: High vs. low	2.4	1.3-3.5	2.2	1.1-3.4	2.2	1.1-3.4
Procedural justice: High vs. low	1.2	0.2-2.3	1.2	0.0-2.4	1.2	0.2-2.3

Supplementary Table S3. Estimated working life expectancies (WLE) at age 50 among Finnish public sector employees of age 50 to 68 (n=70, 662), for job control and job demands by sex and occupational group. [CI= confidence interval; Q=quartile.)

	**Men**						**Women**					
	**Managers and specialized professionals**		**Non-manual professionals**		**Service and manual workers**		**Managers and specialized professionals**		**Non-manual professionals**		**Service and manual workers**	
	**WLE**	**95% CI**	**WLE**	**95% CI**	**WLE**	**95% CI**	**WLE**	**95% CI**	**WLE**	**95% CI**	**WLE**	**95% CI**
**Job control**												
Q1 (High)	13.7	13.6-13.8	13.5	13.4-13.6	13.1	13.0-13.2	13.7	13.6-13.8	13.5	13.4-13.5	13.1	13.0-13.2
Q2	13.5	13.5-13.6	13.3	13.2-13.4	12.9	12.8-13.0	13.5	13.5-13.6	13.3	13.2-13.4	12.9	12.9-13.0
Q3	13.4	13.3-13.5	13.2	13.1-13.3	12.8	12.7-12.9	13.4	13.4-13.5	13.2	13.1-13.3	12.8	12.8-12.9
Q4 (Low)	13.2	13.1-13.3	12.9	12.9-13.0	12.6	12.5-12.7	13.2	13.1-13.2	12.9	12.9-13.0	12.6	12.5-12.6
**Job demands**												
Q1 (Low)	13.7	13.6-13.7	13.3	13.2-13.3	12.8	12.7-12.9	13.7	13.6-13.7	13.3	13.2-13.3	12.8	12.8-12.9
Q2	13.6	13.5-13.7	13.2	13.1-13.3	12.8	12.7-12.9	13.6	13.5-13.7	13.2	13.2-13.3	12.8	12.7-12.9
Q3	13.6	13.5-13.7	13.2	13.1-13.3	12.8	12.7-12.9	13.6	13.5-13.7	13.2	13.1-13.3	12.8	12.7-12.8
Q4 (High)	13.5	13.4-13.6	13.1	13.0-13.2	12.7	12.6-12.8	13.5	13.4-13.5	13.1	13.0-13.2	12.7	12.6-12.7
